# Supplementary material for: Breaking barriers: Validation of a Spanish oral health knowledge tool to enhance patient-provider communication
Source: PLoS One. 2026 Jun 5;21(6):e0350384. doi: 10.1371/journal.pone.0350384 (PMC13241004; doi:10.1371/journal.pone.0350384)
Supplement: Attachment — Submitted filename: PLOS one responses KS.docx [file pone.0350384.s001.docx]

We would like to thank the reviewers for their conscientious and helpful comments.

We address them all here, and the revised version of the manuscript.

Thank you very much for the insightful review.

5. Review Comments to the Author. Please use the space provided to explain your answers to the questions above. You may also include additional comments for the author, including concerns about dual publication, research ethics, or publication ethics. (Please upload your review as an attachment if it exceeds 20,000 characters)

Reviewer #1: Dear authors, Some comments and suggestions for further clarification are listed below.

1- Introduction:

a. Add related studies.

We expanded the background information and added all relevant studies. Please refer to citations 8-16

b. Mention the knowledge gaps of the previous related studies and highlight the importance of your research

We addressed this issue in the paragraph starting at 112

c. Write the hypothesis or null hypothesis

Refer to the statement starting at 126

d. Clarify why open-ended oral questions are suitable for low-literacy populations

Collecting the patient’s own words was critical to the development of the KROHL scale, but also the use of open-ended questions is a recommended strategy for communication among individuals with low literacy.

2- Results:

a. Tables are missing from the manuscript.

All the tables are now included.

3- Discussion:

a. Highlight the advantages of KROHL-S over other tools (oral administration, literacy independence).

Please refer to the paragraph starting at 278

b. Mention limitations (manual scoring, selection bias, generalizability)

Limitations are presented in the paragraph starting at 302

4- Conclusion

a. Emphasize practical applications in clinical practice and patient education.

Please refer to the paragraph starting at 325

b. Highlight its contribution to addressing oral health disparities

Please refer to 329

5- Comprehensive English editing is needed

It was addressed

Good Luck with your submission

Reviewer #2:

The article complies with PLOS One standards. The study was well conducted, concise and objective. Also, it is relevant due to the high number of Spanish-speaking people living in the United States. Suggestions were made below in order to contribute to the publication.

Summary: The abstract is well structured and with all the information necessary to understand the study.

Introduction. On lines 84, 85 and 86: “Research consistently demonstrates a strong correlation between limited health literacy and poor oral health outcomes.” Cite the reference for this statement.

The references are now included 2,6

On lines 91 to 94: “Health literacy not only affects individual knowledge, attitudes and behaviors but also shapes skills related to disease prevention and self-management. In the clinical setting, health knowledge, a key component of health literacy, serves as an effective measure and it is widely used to assess public health interventions.” Cite the reference used for this statement.

Please refer to the paragraphs starting at 107 and 112.

At the end of the introduction, there is not a paragraph on the importance of validating this questionnaire for the Hispanic population. What contributions can this survey on oral health knowledge among Spanish-speaking adults bring to these people living in the United States?

Please refer to the paragraph starting at 126. (also 85 and 112)

Methodology It is written objectively and with all the information necessary for the reproduction of the study.

Discussion On lines 245 and 246: “In contrast to other oral health literacy measures, KROHL-S evaluates condition-specific and domain of knowledge specific aspects of the most common oral conditions.” Cite the reference that supported this statement.

Please refer to the paragraph starting at 112 where we discussed other tools and the statements based on citations 11 and 21.

On lines 272 to 274: “To address this issue, we are currently in the process of incorporation different technologies to capture the responses using voice-to-text applications, as well as training different AIs to automate the scoring.” Explain what these technologies are to capture the responses using voice-to-text applications, as well as training different AIs to automate the scoring, in addition to the types of studies that are being developed.

Please refer to the paragraph starting at 314

Reviewer #3: Dear Authors,I have read your manuscript with interest and would like to offer the following comments.

General comment

I note that you have cited only 13 references. Although this journal does not set a minimum number of citations, I consider that the bibliography should be expanded, particularly in the Introduction and Discussion. Additional references to previous instruments, reporting guidelines, and relevant methodological sources would strengthen the rationale and lend greater weight to the claims made in the manuscript.

We are now providing a more in-depth background information leading to the creation of KROHL, including our previous steps starting at 90. The number of relevant citations is now 36.

Introduction

The manuscript conflates two related but non-equivalent concepts, which may create confusion about what exactly the KROHL-S measures and what conclusions can reasonably be drawn. It would therefore be helpful to delineate the construct more clearly: oral health knowledge versus oral health literacy. While KROHL-S is presented as a knowledge tool, much of the framing draws on the oral health literacy literature. A clearer conceptual framework is recommended, including explicit justification for focusing on knowledge (rather than other literacy dimensions), as the current framing—grounded in health literacy and inequities—may lead readers to infer that the instrument measures oral health literacy more broadly.

This point was addressed in the paragraph starting at 107

I also perceive a gap regarding Spanish-language instruments. It would be useful to clarify which prior instruments exist in Spanish (if any), why they do not capture the intended construct or open-ended format, and what KROHL-S adds compared with available alternatives.

Available tools are discussed in the paragraph starting at 98

Methods

As data collection appears to have been conducted at a single centre, there is potential sampling bias. This should be explicitly acknowledged as a limitation in the Methods section as well.

Please refer to the paragraph starting at 305.

No formal reliability reporting is provided to document how consensus between raters was established (e.g., ICC/kappa,double-scoring of a subsample). Given the open-ended nature of the responses and the scoring procedure, this information is important. In addition, no a priori sample size calculation or formal sample size rationale is presented

ICC results are now reported. Please refer to 245

As the study includes psychometric analyses (including CFA), it would be important to add an explicit “Sample size determination/rationale “section stating whether the final sample (n=177) resulted from convenience sampling and, if so, justifying its adequacy in relation to model complexity (number of factors/items and estimated parameters), the estimator used, and the handling of missing data.

This information is now available. Please refer to 214

Results

Regarding interpretation of the total score: if the maximum score is 38, a mean of 8.34 suggests a possible floor effect. I would recommend providing the score distribution (e.g., histogram), the proportion of zero/low scores by domain, and item-level analyses (difficulty; floor/ceiling effects).

The histogram information for each subscale and the overall KROHL is now reported, starting at 229. Since the concept of a right answer is not applicable to this instrument, item difficulty was not calculated.

With respect to the CFA, the manuscript should avoid characterising model fit as “adequate” without qualification. Values of CFI=0.86 and TLI=0.83 are typically below commonly used thresholds, and RMSEA is statistically significant (p=0.044). These results should be reported in greater detail and interpreted consistently with the Discussion, avoiding overstatement where possible.

The language was removed.

Please also report effect sizes and ideally 95% confidence intervals, and consider adjustment for relevant covariates (e.g., age, years in the USA, education/literacy, insurance status).

We have included confidence intervals where appropriate and show all in Table 3.

Discussion:

The tone of the psychometric conclusions should be moderated, as the manuscript itself notes that “the fit of the overall scale structure was average”. The final summary and the Abstract should therefore avoid presenting the CFA results as “adequate” without nuance.

These have been removed.

The manuscript mentions the possibility of voice-to-text and “training different AIs to automate scoring” as future work. It would be helpful to state explicitly that this was not evaluated in the present study; what validation would be required (e.g., transcription error, dialect-related bias, comparison against human raters, privacy considerations); and that any automation would necessitate a new study of validity and reliability.

Please review the paragraph starting at 314

Conclusions

Please avoid generalisations beyond the sample (single-centre, convenience sampling). In addition, the conclusions should be aligned with the CFA results (i.e., average/moderate fit).

Please review the conclusion starting at 328

Data repository PLOS ONE requires a Data Availability Statement and that the underlying data be available without restriction. The manuscript indicates “available on request”, which is not acceptable under the journal’s policy.

The data is now available through the following link: [https://doi.org/10.58153/frv59-hzf81](https://urldefense.proofpoint.com/v2/url?u=https-3A__doi.org_10.58153_frv59-2Dhzf81&d=DwMFaQ&c=slrrB7dE8n7gBJbeO0g-IQ&r=Gz9scghUPKeS3fttVbzMlXSP3u1ydznNTIKneq5_ExE&m=tj62FEoOsoCUHUdK8vgdXUAbV7f-e4tcrJ76dfg7HOhtMiPnJvn-pwbLQoB2i-yL&s=57d0AIQyM7WCf8kiEgKwLh_XuA1n0IQtEoIzDk51mAU&e=)

6. PLOS authors have the option to publish the peer review history of their article (what does this mean?). If published,this will include your full peer review and any attached files.If you choose “no”, your identity will remain anonymous but your review may still be made public.**Do you want your identity to be public for this peer review?** For information about this choice, including consentwithdrawal, please see our Privacy Policy.

Reviewer #1: No

Reviewer #2: No

Reviewer #3: No

[NOTE: If reviewer comments were submitted as an attachment file, they will be attached to this email and accessible viathe submission site. Please log into your account, locate the manuscript record, and check for the action link "ViewAttachments". If this link does not appear, there are no attachment files.]

To ensure your figures meet our technical requirements, please review our figure guidelines: https://journals.plos.org/plosone/s/figures

You may also use PLOS’s free figure tool, NAAS, to help you prepare publication quality figures: https://journals.plos.org/plosone/s/figures#loc-tools-for-figure-preparation.

NAAS will assess whether your figures meet our technical requirements by comparing each figure against our figure specifications.
