# Supplementary material for: Breaking barriers: Validation of a Spanish oral health knowledge tool to enhance patient-provider communication
Source: PLoS One. 2026 Jun 5;21(6):e0350384. doi: 10.1371/journal.pone.0350384 (PMC13241004; doi:10.1371/journal.pone.0350384)
Supplement: Attachment — Submitted filename: Responses 2.04.19.docx [file pone.0350384.s002.docx]

1. No formal reliability reporting is provided to document how consensus between raters was established (e.g., ICC/kappa,double-scoring of a subsample).

In our previous revision, we included the ICC to assess interrater reliability (see lines 209, 245-246).

2. In this comment: "Internal consistency was good for the general scale and for the oral cancer and malocclusion subscales, and acceptable for dental caries and periodontal disease." It is necessary to include the values Internal consistency".

These were in table 2, but we forgot to add them directly in-line. We’ve done so in our revision.

3. In addition, it is necessary that the research has not been published in any journal before and complies with the ethical principles of research with human beings.

This information was already provided during submission as it is required by publisher.

4. First, although a “Sample Size Rationale” section has been added, the justification provided for the adequacy of the sample in relation to the confirmatory factor analysis remains too general. A more explicit explanation linking the final sample size to the complexity of the tested model would be necessary to support the robustness of the CFA results.

We’ve added further discussion on these limitations (lines 313-317)

5. Second, there is still an inconsistency in the interpretation of model fit. While the Discussion adopts a more cautious tone, the Abstract continues to describe the CFA results as indicating “adequate fit.” Given the reported indices, this wording appears stronger than what the data support. The interpretation of model fit should be aligned throughout the manuscript to ensure internal consistency and appropriate methodological caution.

Thank you for catching this, this has been revised to be consistent throughout.
